# Improving Functional Abilities in Children and Adolescents with Autism Spectrum Disorder Using Non-Invasive REAC Neuro Psycho Physical Optimization Treatments: A PEDI-CAT Study

**DOI:** 10.3390/jpm13050792

**Published:** 2023-05-05

**Authors:** Arianna Rinaldi, Márcia C. Marins Martins, Ana C. De Almeida Martins Oliveira, Salvatore Rinaldi, Vania Fontani

**Affiliations:** 1 Department of Biomedical Sciences, University of Sassari, 07100 Sassari, Italy; 2 Department of Adaptive Neuro Psycho Physio Pathology and Neuro Psycho Physical Optimization, Rinaldi Fontani Institute, 50144 Florence, Italy; 3 Research Department, Rinaldi Fontani Foundation, 50144 Florence, Italy; 4 International Scientific Society of Neuro Psycho Physical Optimization with REAC Technology, Brazilian Branch, Sao Paulo 01000-000, Brazil

**Keywords:** autism spectrum disorder, endogenous bioelectric activity, epigenetic, radioelectric asymmetric conveyer technology, neurobiological stimulation, neurobiological modulation, PEDI-CAT

## Abstract

Autism Spectrum Disorder (ASD) is a complex neurodevelopmental disorder that affects communication, social interaction, and behavior. Non-invasive neuromodulation techniques, such as radioelectric asymmetric conveyer (REAC) technology, have gained attention for their potential to improve the endogenous bioelectric activity (EBA) and neurobiological processes underlying ASD. Neuro Postural Optimization (NPO) and Neuro Psycho Physical Optimization (NPPO) treatments are non-invasive and painless neuromodulation treatments that utilize REAC technology and have shown promising results in improving the symptoms of ASD. This study aimed to evaluate the effects of NPO and NPPO treatments on functional abilities in children and adolescents with ASD using the Pediatric Evaluation of Disability Inventory-Computer Adaptive Test (PEDI-CAT). The study consisted of 27 children and adolescents with ASD who underwent a single session of NPO followed by 18 sessions of NPPO treatment over a period of one week. The results showed significant improvements in the children’s and adolescents’ functional abilities across all domains of the PEDI-CAT. These findings suggest that NPO and NPPO may be effective treatments for improving functional abilities in children and adolescents with ASD.

## 1. Introduction

Autism Spectrum Disorder (ASD) is a neurodevelopmental condition [1] characterized by difficulties in social interaction, communication, and restricted, repetitive patterns of behavior. This heterogeneous disorder exhibits varying symptoms among individuals. The precise cause of ASD remains unknown, although recent research suggests that a combination of genetic and environmental factors influence the development of the disorder [1].

Behavioral, mood, and psychiatric disorders are prevalent in individuals with ASD [2], which can significantly impair social, academic, and occupational functioning. Commonly observed behavioral disorders include attention-deficit/hyperactivity disorder (ADHD) [3] and obsessive-compulsive disorder (OCD), with a prevalence rate of up to 60% and 30% [4], respectively. Mood disorders, such as anxiety and depression, are also common in individuals with ASD, with a prevalence rate of up to 30% and 50%, respectively [5,6]. Psychiatric disorders such as schizophrenia and bipolar disorder are less common, with a prevalence rate of up to 1% and 4%, respectively [7,8]. However, individuals with ASD have a higher risk of developing these disorders compared to the general population [9]. The presence of these co-occurring disorders can complicate diagnosis and treatment, leading to poorer outcomes for individuals with ASD [10].

Epigenetic factors have also been implicated in the development of ASD and its co-occurring disorders [11]. Environmental factors such as exposure to toxins or stress can influence epigenetic changes. Epigenetic modifications can affect the expression of genes involved in brain development and function, including those associated with ASD [12]. For instance, DNA methylation changes in the oxytocin receptor gene have been linked to social deficits in individuals with ASD [12]. Similarly, alterations in gene expression involved in regulating the stress response have been linked to anxiety and depression in individuals with ASD [13,14].

There is no cure for autism, but various treatments are available that aim to improve the symptoms and quality of life of individuals with autism [15].

Among the various interventions available, non-invasive neuromodulation techniques are gaining attention due to their potential for improving the neurobiological processes underlying the disorder. Radioelectric asymmetric conveyer (REAC) technology is a non-invasive neuromodulation technique that has been proposed as a potential intervention for ASD [16,17]. REAC technology treatment is a non-invasive, safe, and effective method of neurobiological modulation that uses radioelectric asymmetric conveyer fields to modulate nervous system functionality.

Neuro Postural Optimization (NPO), Neuro Psycho Physical Optimization (NPPO), and Neuro Psycho Physical Optimization-Cervico Brachial (NPPO-CB) are REAC neuromodulation treatments. NPO aims to optimize the posture balance and motor control of the individual [18,19], while NPPO aims to improve mood and behavioral, cognitive, and emotional processing [20,21]. Both techniques are non-invasive and painless and have shown promising results in improving the symptoms of ASD [16,17]. The purpose of this paper is to present the findings of a study that analyzed the effects of NPO and NPPO treatments on children and adolescents with ASD using the PEDI-CAT [22,23,24]. The study included 27 children and adolescents with ASD who underwent a session of NPO and 18 sessions of NPPO treatment over one week. The results of the study showed improvements in the children’s and adolescents’ functional abilities across all domains of the PEDI-CAT.

## 2. Materials and Methods

### 2.1. Sample Size Determination and Power Analysis

We conducted a power analysis with G*power (Universität Düsseldorf, Psychologie—HHU) [25], assuming a large effect size of 0.80, an alpha error probability of 0.05, and a desired power of 0.95 on a given sample size.

The analysis indicated that a total sample size of 20 participants would be sufficient for our study. However, due to our compassionate approach, we decided to include all candidates who met our inclusion criteria instead of limiting it to the required sample size of 20.

### 2.2. Inclusion Criteria

Boys and girls aged 3 to 21 years, with a prior diagnosis of autism confirmed by Autism Diagnostic Interview-Revised (ADI-R) and functional abilities comparable to those of a child between 6 months and 7 years of age, whose parents expressly requested to receive REAC neuromodulation treatments and participate in the psychometric test were included.

### 2.3. Population

The study comprised 27 children and adolescents between 3 and 17 years of age, consisting of 24 males, mean age: 6.71, and 3 females, mean age: 5.67.

### 2.4. Research Locations

The study was conducted in a Clinic affiliated with the International Scientific Society of Neuro Psycho-Physical Optimization with REAC Technology, Brazilian Branch, Brazil, and at the Rinaldi Fontani Institute and Foundation, Italy.

### 2.5. Assessments

In this study, two assessments were conducted: the evaluation of functional dysmetria [26,27] and the administration of the PEDI-CAT questionnaire [23]. These assessments were performed both before and after the administration of REAC NPO and NPPO treatments, with a follow-up conducted approximately 3–4 months after the treatments. In order to assess the immediate response to the NPO treatment, the disappearance of functional dysmetria was verified immediately after its administration. At the follow-up checkup, stable correction of functional dysmetria was observed, and a second administration of the PEDI-CAT [22,23,24] questionnaire was conducted.

### 2.6. Functional Dysmetria

Functional dysmetria (FD) is a neuro psycho motor expression of adaptive dysfunction characterized by a maladaptive response to internal or external stressors [27]. It results in an alteration of the processes that coordinate the execution of voluntary movements. The FD can occur in healthy individuals [18] and is not associated with structural or neurological damage.

### 2.7. Pediatric Evaluation of Disability Inventory-Computer Adaptive Test (PEDI-CAT)

The PEDI-CAT is a comprehensive assessment tool used to evaluate functional skills and abilities in children and adolescents with disabilities [22,23]. The PEDI-CAT assesses the developmental milestones of children and adolescents aged from 2 to 21 years across four subscales: activities of daily living, mobility, responsibility, and social cognition. It also assesses the consistency of caregiver responses to reduce potential biases in caregiver-filled tests. The test takes approximately 20–30 min to complete and can be administered by a trained healthcare professional or therapist.

One of the conditions for which the PEDI-CAT is frequently used is ASD [24]. Children and adolescents with ASD often experience challenges with daily living skills such as self-care, mobility, and socialization. The PEDI-CAT is specifically designed to evaluate a child’s abilities in these areas and identify areas of strength and weakness.

The PEDI-CAT has been validated for use in children and adolescents with ASD [24], demonstrating reliability and validity in assessing functional skills in this population. The assessment results provide valuable information about a child’s functional abilities, which can be used to develop individualized treatment plans, monitor progress over time, and inform educational and therapeutic interventions.

### 2.8. Neuro Postural Optimization (NPO)

The REAC Neuro Postural Optimization (NPO) treatment is a one-session neurobiological modulation technique that aims to optimize endogenous bioelectric activity (EBA) and cerebral electro-metabolic activity, resulting in long-lasting greater efficiency and functionality compared to previous modifications induced by dysfunctional adaptations [18,27,28]. The primary clinical outcome of NPO treatment is an immediate and long-lasting correction/disappearance of functional dysmetria and sustained improvement in motor strategies and balance [19,29].

### 2.9. Neuro Psycho Physical Optimization Treatments

The REAC Neuro Psycho Physical Optimization (NPPO) treatments are neurobiological modulation treatments designed to optimize EBA to enhance neuro-psycho-physical performance, resulting in improved mood, behavior, cognitive and emotional control, physical performance, and overall well-being [20,21,30,31]. This approach has also been applied to individuals with ASD [16,17,29].

### 2.10. Statistics

For the statistical evaluation of the study, the G*Power (version 3.1.9.7) and SPSS 22 software (Version 22) were used. The Wilcoxon method was employed to assess sample size and statistical significance across all values obtained in the research.

The study was conducted in accordance with the Declaration of Helsinki and approved by the Institutional Review Board of the Center for Developmental Biology and Reprogramming of the University of Sassari, Italy, approval code N-02/21.

## 3. Results

### 3.1. NPO and Functional Dysmetria

Administration of the NPO treatment resulted in an immediate correction of the FD, which was subsequently confirmed at the 3–4 month follow-up assessment.

### 3.2. PEDI-CAT

#### 3.2.1. Activities of Daily Living

The assessment of the Daily Activities domain in PEDI-CAT encompasses a total of 68 elements that are distributed among 4 distinct content domains, namely Getting Dressed, Keeping Clean, Home Tasks, and Eating and Mealtime. Each of these content areas falls under the umbrella of the Daily Activities domain. The assessment of children’s and adolescents’ abilities is conducted using a 4-point Difficulty Scale, where the response options vary from unable to easy. The results showed a significant statistical difference at a significance level of *p* < 0.5.

**Figure 1:**
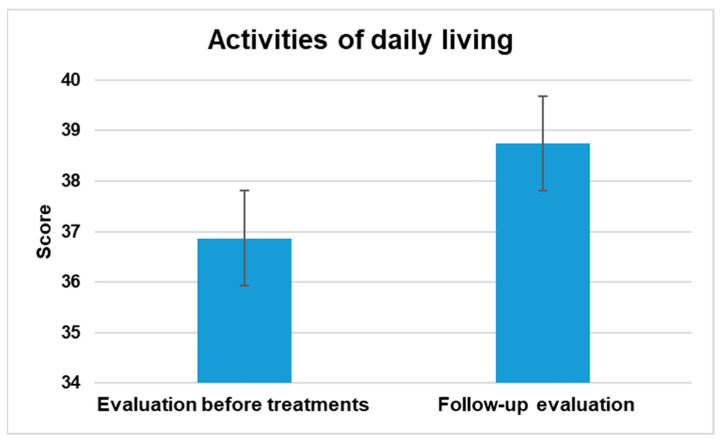
The figure displays the general enhancement in the activities of the daily living domain assessed between the average scores prior to interventions and during the follow-up evaluation.

Figure 1 illustrates the difference in mean scores before treatment and at follow-up, indicating overall improvement.

#### 3.2.2. Mobility

The PEDI-CAT Mobility domain comprises 75 items distributed across 4 content areas, namely Basic Movement and Transfers, Standing and Walking, Steps and Inclines, and Running and Playing. The assessment of children’s and adolescents’ abilities is conducted using a 4-point Difficulty Scale, where the response options vary from unable to easy. The results showed a significant statistical difference at a significance level of *p* < 0.5.

**Figure 2:**
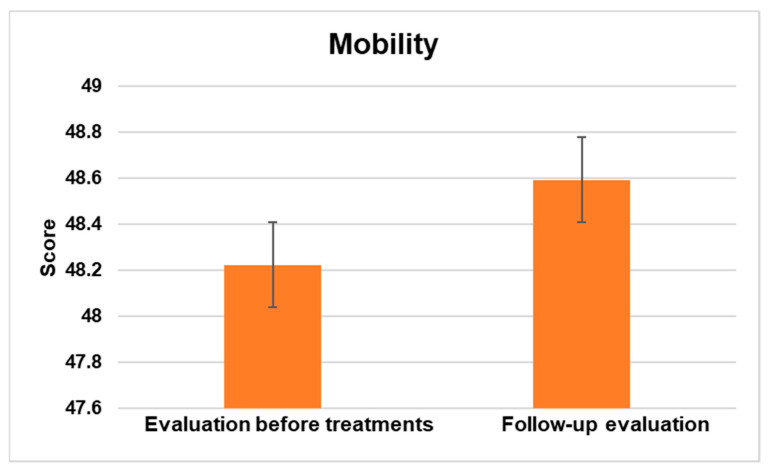
The figure displays the general enhancement in the mobility domain assessed between the average scores prior to interventions and during the follow-up evaluation.

Figure 2 illustrates the overall enhancement assessed between the mean scores prior to interventions and during the follow-up period.

#### 3.2.3. Social Cognition

The Social/Cognitive domain of the PEDI-CAT constituted 60 items in 4 content areas of Interaction, Communication, Everyday Cognition, and Self-Management. The assessment of children’s and adolescents’ abilities is conducted using a 4-point Difficulty Scale, where the response options vary from unable to easy. The results showed a significant statistical difference at a significance level of *p* < 0.5.

**Figure 3:**
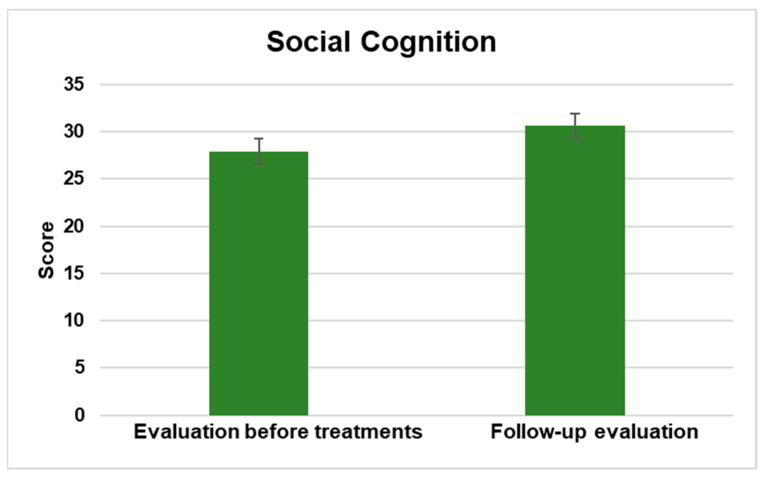
The figure displays the general enhancement in the social cognition domain assessed between the average scores prior to interventions and during the follow-up evaluation.

Figure 3 displays the enhancement observed in the mean scores from pre-treatment to follow-up evaluation.

#### 3.2.4. Responsibility

The Responsibility domain of the PEDI-CAT comprises 51 items that evaluate a young person’s ability to manage life tasks necessary for independent living, categorized into 4 content areas: Organization and Planning, Taking Care of Daily Needs, Health Management, and Staying Safe. This domain requires the utilization of multiple functional skills in combination to carry out life tasks, rendering it a more challenging area. As a result, it is assessed in children and adolescents aged 3 to 21 years. The Responsibility domain encompasses a distinct 5-point Responsibility Scale, comprising responses that range from Adult/caregiver has complete responsibility, the child assumes no responsibility to the child independently takes full responsibility without any form of direction, supervision, or guidance from an adult/caregiver. The results showed a significant statistical difference at a significance level of *p* < 0.5.

**Figure 4:**
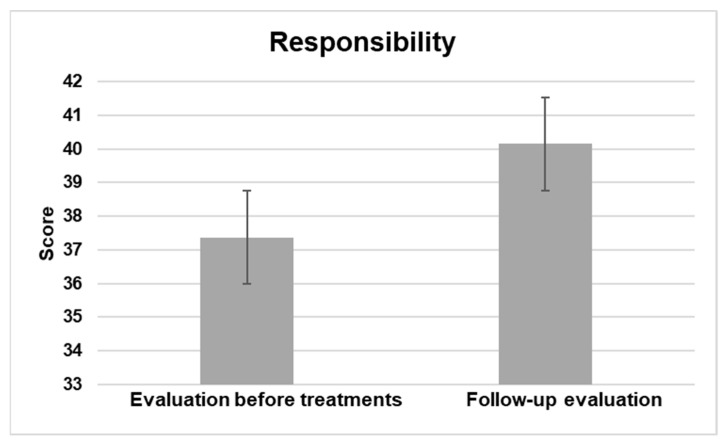
The figure displays the general enhancement in the responsibility domain assessed between the average scores prior to interventions and during the follow-up evaluation.

Figure 4 displays the general enhancement assessed between the average scores prior to interventions and during the follow-up evaluation.

## 4. Discussion

ASD is a highly prevalent and complex neurodevelopmental disorder with significant heterogeneity in presentation, etiology, and treatment response. The pathogenesis of ASD is thought to involve complex interactions between genetic, epigenetic, environmental, and neurobiological factors [32,33,34], whose common origin is an alteration of endogenous bioelectrical activity (EBA) [35,36,37].

The study of EBA and its role in neurobiology has garnered increasing attention in recent years due to its involvement in fundamental processes such as neurotransmission and epigenetics [38], as well as its potential implications for disorders such as ASD [39]. In this discussion, we explored the current understanding of EBA in neurobiology and its relevance to ASD to gain a deeper understanding of how the REAC NPO and NPPO treatments, which have been studied to improve EBA in neurotransmission processes, influenced the results presented in this manuscript.

EBA plays a critical role in various physiological processes, including wound healing, cell migration, and tissue regeneration [40]. In the brain, EBA has been shown to modulate neuronal activity, regulate synaptic plasticity, and influence the formation and maintenance of neural circuits and neurotransmission [41,42].

EBA also plays a crucial role in epigenetics [37,43], the study of heritable changes in gene expression that do not involve alterations to the underlying DNA sequence [44,45]. One of the ways EBA affects gene expression is by influencing the methylation of the DNA [43]. Methylation is a process by which methyl groups are added to the DNA molecule, affecting its accessibility to transcription factors and thereby regulating gene expression [46].

The connection between EBA and ASD has been a subject of increasing interest in recent years. Studies have shown that individuals with ASD have altered brain connectivity, with abnormalities in synaptic transmission and plasticity [47,48,49].

Therefore, it is possible that EBA alterations may contribute to the development of ASD by affecting gene expression and neuronal connectivity.

REAC technology uses asymmetrically conveyed radioelectric fields to optimize EBA at different levels of the organization. By considering the essential role of EBA in epigenetic processes, neuroplasticity, and neurotransmission, we can infer that REAC treatments aimed at optimizing EBA across multiple levels represent a potential technological approach to precision medicine, particularly in the context of ASD.

REAC NPO and NPPO treatments are non-invasive and painless neuromodulation treatments that optimize EBA levels in neurotransmission processes and have shown promising results in improving the symptoms of ASD [16,17].

These treatments target the neurobiological and postural imbalances that are common in individuals with ASD, potentially leading to improvements in functional abilities.

This study aimed to evaluate the effects of NPO and NPPO treatments on functional abilities in children and adolescents with ASD using the PEDI-CAT. The results showed significant improvements across all domains. In the activities of the Daily Living Domain, which include Getting Dressed, Keeping Clean, Home Tasks, and Eating and Mealtime, there was a statistically significant improvement with a *p* < 0.5. The Mobility domain, which comprises Basic Movement and Transfers, Standing and Walking, Steps and Inclines, and Running and Playing, showed significant improvement with a *p* < 0.5. Similarly, there was a significant improvement in the Social Cognition domain, which includes Interaction, Communication, Everyday Cognition, and Self-Management, with a *p* < 0.5. Finally, in the Responsibility domain, which assesses a child’s ability to manage life tasks necessary for independent living, there was also a significant improvement with a *p* < 0.5. The results indicate that NPO and NPPO could be effective treatments for enhancing the functional abilities of children and adolescents with ASD.

These findings suggest that REAC NPO and NPPO treatments have potential applications in precision medicine approaches for ASD by optimizing individual EBA alterations that underlie disrupted neurotransmission processes and epigenetic profiles. However, further research is needed to better understand the mechanisms of EBA alteration and the effects of REAC technology on this mechanism, particularly with respect to neurotransmission disorders and epigenetic dysregulation in ASD.

## 5. Conclusions

In conclusion, this manuscript highlights the growing understanding of the role of EBA in neurobiology and its potential implications for neurodevelopmental disorders such as ASD. The complex interactions between genetic, epigenetic, environmental, and neurobiological factors in the pathogenesis of ASD are believed to be linked to alterations in EBA [41,50,51]. The use of REAC NPO and NPPO treatments, which aim to optimize EBA in neurotransmission processes, has shown promising results in improving functional abilities in children and adolescents with ASD [16,17].

The critical role of EBA in various physiological processes, including synaptic plasticity and epigenetics [51,52], underscores the potential of REAC technology as a precision medicine approach for ASD. The significant improvements observed in multiple domains of functional abilities, as assessed by the PEDI-CAT, suggest that REAC NPO and NPPO treatments could be effective in enhancing the daily living skills, mobility, social cognition, and responsibility of individuals with ASD.

The limitations of this study are typical of an open-label study. However, since the effectiveness of REAC neuromodulation treatments aimed at improving mood and behavioral disorders in developmental age, used in this study, has been extensively confirmed by previous studies, the limitations of the open-label design are significantly reduced. However, further research is needed to better understand the mechanisms of EBA alterations and the effects of REAC neurobiological treatments on these mechanisms, particularly in the context of neurotransmission disorders and epigenetic dysregulation in ASD.

Nevertheless, the findings presented in this manuscript support the potential applications of REAC NPO and NPPO treatments as novel and non-invasive approaches in precision medicine for individuals with ASD, with the goal of optimizing individual EBA alterations to improve functional outcomes [16,17].

## Data Availability

All data is contained within the manuscript.

## References

[1] Kumar S., Reynolds K., Ji Y., Gu R., Rai S., Zhou C.J. (2019). Impaired neurodevelopmental pathways in autism spectrum disorder: A review of signaling mechanisms and crosstalk. J. Neurodev. Disord..

[2] Kaat A.J., Lecavalier L. (2013). Disruptive behavior disorders in children and adolescents with autism spectrum disorders: A review of the prevalence, presentation, and treatment. Res. Autism Spectr. Disord..

[3] Schachar R.J., Dupuis A., Arnold P.D., Anagnostou E., Kelley E., Georgiades S., Nicolson R., Townes P., Burton C.L., Crosbie J. (2023). Autism Spectrum Disorder and Attention-Deficit/Hyperactivity Disorder: Shared or Unique Neurocognitive Profiles?. Res. Child Adolesc. Psychopathol..

[4] Pazuniak M., Pekrul S.R. (2020). Obsessive–Compulsive Disorder in Autism Spectrum Disorder Across the Lifespan. Child Adolesc. Psychiatr. Clin. N. Am..

[5] Hudson C.C., Hall L., Harkness K.L. (2019). Prevalence of Depressive Disorders in Individuals with Autism Spectrum Disorder: A Meta-Analysis. J. Abnorm. Child Psychol..

[6] Hollocks M.J., Lerh J.W., Magiati I., Meiser-Stedman R., Brugha T.S. (2019). Anxiety and depression in adults with autism spectrum disorder: A systematic review and meta-analysis. Psychol. Med..

[7] Canitano R., Pallagrosi M. (2017). Autism Spectrum Disorders and Schizophrenia Spectrum Disorders: Excitation/Inhibition Imbalance and Developmental Trajectories. Front. Psychiatry.

[8] Carroll L.S., Owen M.J. (2009). Genetic overlap between autism, schizophrenia and bipolar disorder. Genome Med..

[9] Vaquerizo-Serrano J., de Pablo G.S., Singh J., Santosh P. (2022). Autism Spectrum Disorder and Clinical High Risk for Psychosis: A Systematic Review and Meta-analysis. J. Autism Dev. Disord..

[10] Mosner M.G., Kinard J.L., Shah J.S., McWeeny S., Greene R.K., Lowery S.C., Mazefsky C.A., Dichter G.S. (2019). Rates of Co-occurring Psychiatric Disorders in Autism Spectrum Disorder Using the Mini International Neuropsychiatric Interview. J. Autism Dev. Disord..

[11] Eshraghi A.A., Liu G., Kay S.-I.S., Eshraghi R.S., Mittal J., Moshiree B., Mittal R. (2018). Epigenetics and Autism Spectrum Disorder: Is There a Correlation?. Front. Cell. Neurosci..

[12] Loke Y.J., Hannan A.J., Craig J.M. (2015). The Role of Epigenetic Change in Autism Spectrum Disorders. Front. Neurol..

[13] Kubota T., Mochizuki K. (2016). Epigenetic Effect of Environmental Factors on Autism Spectrum Disorders. Int. J. Environ. Res. Public Health.

[14] Khogeer A.A., AboMansour I.S., Mohammed D.A. (2022). The Role of Genetics, Epigenetics, and the Environment in ASD: A Mini Review. Epigenomes.

[15] Frye R.E. (2022). A Personalized Multidisciplinary Approach to Evaluating and Treating Autism Spectrum Disorder. J. Pers. Med..

[16] Rinaldi A., Maioli M., Martins M.C.M., de Castro P.C.F., Silva N.A.P.d.O., de Mattos J.A.V., Fontani V., Rinaldi S. (2021). REAC Non-invasive Neurobiological Stimulation for Mitigating the Impact of Internalizing Disorders in Autism Spectrum Disorder. Adv. Neurodev. Disord..

[17] Rinaldi A., Martins M.C.M., Maioli M., Rinaldi S., Fontani V. (2022). REAC Noninvasive Neurobiological Stimulation in Autism Spectrum Disorder for Alleviating Stress Impact. Adv. Neurodev. Disord..

[18] Rinaldi S., Mura M., Castagna A., Fontani V. (2014). Long-lasting changes in brain activation induced by a single REAC technology pulse in Wi-Fi bands. Randomized double-blind fMRI qualitative study. Sci. Rep..

[19] Rinaldi S., Fontani V., Castagna A., Lotti M. (2012). Noninvasive radioelectric asymmetric conveyor brain stimulation treatment improves balance in individuals over 65 suffering from neurological diseases: Pilot study. Ther. Clin. Risk Manag..

[20] Barcessat A.R.P., Bittencourt M.N., Ferreira L.D., Neri E.D.S., Pereira J.A.C., Bechelli F., Rinaldi A. (2020). REAC Cervicobrachial Neuromodulation Treatment of Depression, Anxiety, and Stress During the COVID-19 Pandemic. Psychol. Res. Behav. Manag..

[21] Rinaldi A., Rinaldi C., Pereira J.A.C., Margotti M.L., Bittencourt M.N., Barcessat A.R.P., Fontani V., Rinaldi S. (2019). Radio electric asymmetric conveyer neuromodulation in depression, anxiety, and stress. Neuropsychiatr. Dis. Treat..

[22] Conroy S., Evans T., Butler-Moburg D., Beuttler R., Robinson J., Huebert M., Mahony E.O., Grant-Beuttler M. (2022). Clinical application and feasibility of utilizing the PEDI-CAT to assess activity and participation among children receiving physical therapy incorporating hippotherapy. Physiother. Theory Pract..

[23] Cordeiro L., Villagomez A., Swain D., Deklotz S., Tartaglia N. (2020). Adaptive Skills in FXS: A Review of the Literature and Evaluation of the PEDI-Computer Adaptive Test (PEDI-CAT) to Measure Adaptive Skills. Brain Sci..

[24] Kramer J.M., Coster W.J., Kao Y.-C., Snow A., Orsmond G.I. (2012). A New Approach to the Measurement of Adaptive Behavior: Development of the PEDI-CAT for Children and Youth with Autism Spectrum Disorders. Phys. Occup. Ther. Pediatr..

[25] Kang H. (2021). Sample size determination and power analysis using the G*Power software. J. Educ. Eval. Health Prof..

[26] Rinaldi S., Mura M., Castagna A., Fontani V. (2012). Preliminary pilot fMRI study of neuropostural optimization with a noninvasive asymmetric radioelectric brain stimulation protocol in functional dysmetria. Neuropsychiatr. Dis. Treat..

[27] Fontani V., Rinaldi A., Rinaldi C., Araldi L., Azzarà A., Carta A.M., Casale N., Castagna A., Del Medico M., Di Stasio M. (2022). Long-Lasting Efficacy of Radio Electric Asymmetric Conveyer Neuromodulation Treatment on Functional Dysmetria, an Adaptive Motor Behavior. Cureus.

[28] Cruz A.V.G.d.O., Gonçalves R.G., Nunes L., de Oliveira J.D.Q., Monteiro E.S.L., Eneias I.S., Lima T.C.G., Ferreira L.D., Neri E.S., Pena J.L.d.C. (2022). Neuro Postural Optimization Neuromodulation Treatment of Radio Electric Asymmetric Conveyer Technology on Stress and Quality of Life in Institutionalized Children in a Capital City of the Brazilian Amazon. Cureus.

[29] Olazarán J., González B., López-Álvarez J., Castagna A., Osa-Ruiz E., Herrero-Cano V., Agüera-Ortiz L., Rinaldi S., Martínez-Martín P. (2013). Motor Effects of REAC in Advanced Alzheimer’s Disease: Results from a Pilot Trial. J. Alzheimer’s Dis..

[30] Rinaldi S., Fontani V., Aravagli L., Lotti M., Castagna A., Mannu P. (2012). Neuropsychophysical optimization by REAC technology in the treatment of: Sense of stress and confusion. Psychometric evaluation in a randomized, single blind, sham-controlled naturalistic study. Patient Prefer. Adherence.

[31] Pereira J.A.C., Rinaldi A., Fontani V., Rinaldi S. (2018). REAC neuromodulation treatments in subjects with severe socioeconomic and cultural hardship in the Brazilian state of Pará: A family observational pilot study. Neuropsychiatr. Dis. Treat..

[32] Alanzi T., Alhashem A., Dagriri K., Alzahrani F., Alkuraya F.S. (2020). A de novo splicing variant supports the candidacy of TLL1 in ASD pathogenesis. Eur. J. Hum. Genet..

[33] Beopoulos A., Géa M., Fasano A., Iris F. (2023). RNA epitranscriptomics dysregulation: A major determinant for significantly increased risk of ASD pathogenesis. Front. Neurosci..

[34] Castora F.J. (2019). Mitochondrial function and abnormalities implicated in the pathogenesis of ASD. Prog. Neuro-Psychopharmacol. Biol. Psychiatry.

[35] Cervera J., Meseguer S., Mafe S. (2016). The interplay between genetic and bioelectrical signaling permits a spatial regionalisation of membrane potentials in model multicellular ensembles. Sci. Rep..

[36] Pietak A., Levin M. (2017). Bioelectric gene and reaction networks: Computational modelling of genetic, biochemical and bioelectrical dynamics in pattern regulation. J. R. Soc. Interface.

[37] Levin M. (2014). Molecular bioelectricity: How endogenous voltage potentials control cell behavior and instruct pattern regulation in vivo. Mol. Biol. Cell.

[38] Roohi-Azizi M., Azimi L., Heysieattalab S., Aamidfar M. (2017). Changes of the brain’s bioelectrical activity in cognition, consciousness, and some mental disorders. Med. J. Islam. Repub. Iran.

[39] Lushchekina E.A., Lushchekin V.S., Strelets V.B. (2018). Bioelectric Brain Activity in Children with Autistic Spectrum Disorders: Population Heterogeneity. Hum. Physiol..

[40] Mathews J., Levin M. (2018). The body electric 2.0: Recent advances in developmental bioelectricity for regenerative and synthetic bioengineering. Curr. Opin. Biotechnol..

[41] Fröhlich F., McCormick D.A. (2010). Endogenous Electric Fields May Guide Neocortical Network Activity. Neuron.

[42] Qiu C., Shivacharan R.S., Zhang M., Durand D.M. (2015). Can Neural Activity Propagate by Endogenous Electrical Field?. J. Neurosci..

[43] Tseng A.-S., Levin M. (2012). Transducing Bioelectric Signals into Epigenetic Pathways During Tadpole Tail Regeneration. Anat. Rec..

[44] Levin M. (2014). Endogenous bioelectrical networks store non-genetic patterning information during development and regeneration. J. Physiol..

[45] Funk R.H.W. (2015). Endogenous electric fields as guiding cue for cell migration. Front. Physiol..

[46] Moore L.D., Le T., Fan G. (2013). DNA Methylation and Its Basic Function. Neuropsychopharmacology.

[47] Guang S., Pang N., Deng X., Yang L., He F., Wu L., Chen C., Yin F., Peng J. (2018). Synaptopathology Involved in Autism Spectrum Disorder. Front. Cell. Neurosci..

[48] Bonsi P., De Jaco A., Fasano L., Gubellini P. (2021). Postsynaptic autism spectrum disorder genes and synaptic dysfunction. Neurobiol. Dis..

[49] Carroll L., Braeutigam S., Dawes J.M., Krsnik Z., Kostovic I., Coutinho E., Dewing J.M., Horton C.A., Gomez-Nicola D., Menassa D.A. (2020). Autism Spectrum Disorders: Multiple Routes to, and Multiple Consequences of, Abnormal Synaptic Function and Connectivity. Neurosci..

[50] Rebollo B., Telenczuk B., Navarro-Guzman A., Destexhe A., Sanchez-Vives M.V. (2021). Modulation of intercolumnar synchronization by endogenous electric fields in cerebral cortex. Sci. Adv..

[51] Shivacharan R.S., Chiang C.-C., Zhang M., Gonzalez-Reyes L.E., Durand D.M. (2019). Self-propagating, non-synaptic epileptiform activity recruits neurons by endogenous electric fields. Exp. Neurol..

[52] Ferreira F., Moreira S., Barriga E.H. (2021). Stretch-induced endogenous electric fields drive neural crest directed collective cell migration in vivo. bioRxiv.

